# Supplementary material for: Targeting colonic macrophages improves glycemic control in high-fat diet-induced obesity
Source: Commun Biol. 2022 Apr 19;5:370. doi: 10.1038/s42003-022-03305-z (PMC9018739; doi:10.1038/s42003-022-03305-z)
Supplement: Supplementary file 2 — Supplementary Information [file 42003_2022_3305_MOESM2_ESM.pdf]

Supplementary Information

Targeting colonic macrophages improves glycemic control in high-fat diet-induced obesity

Supplementary Fig. 1

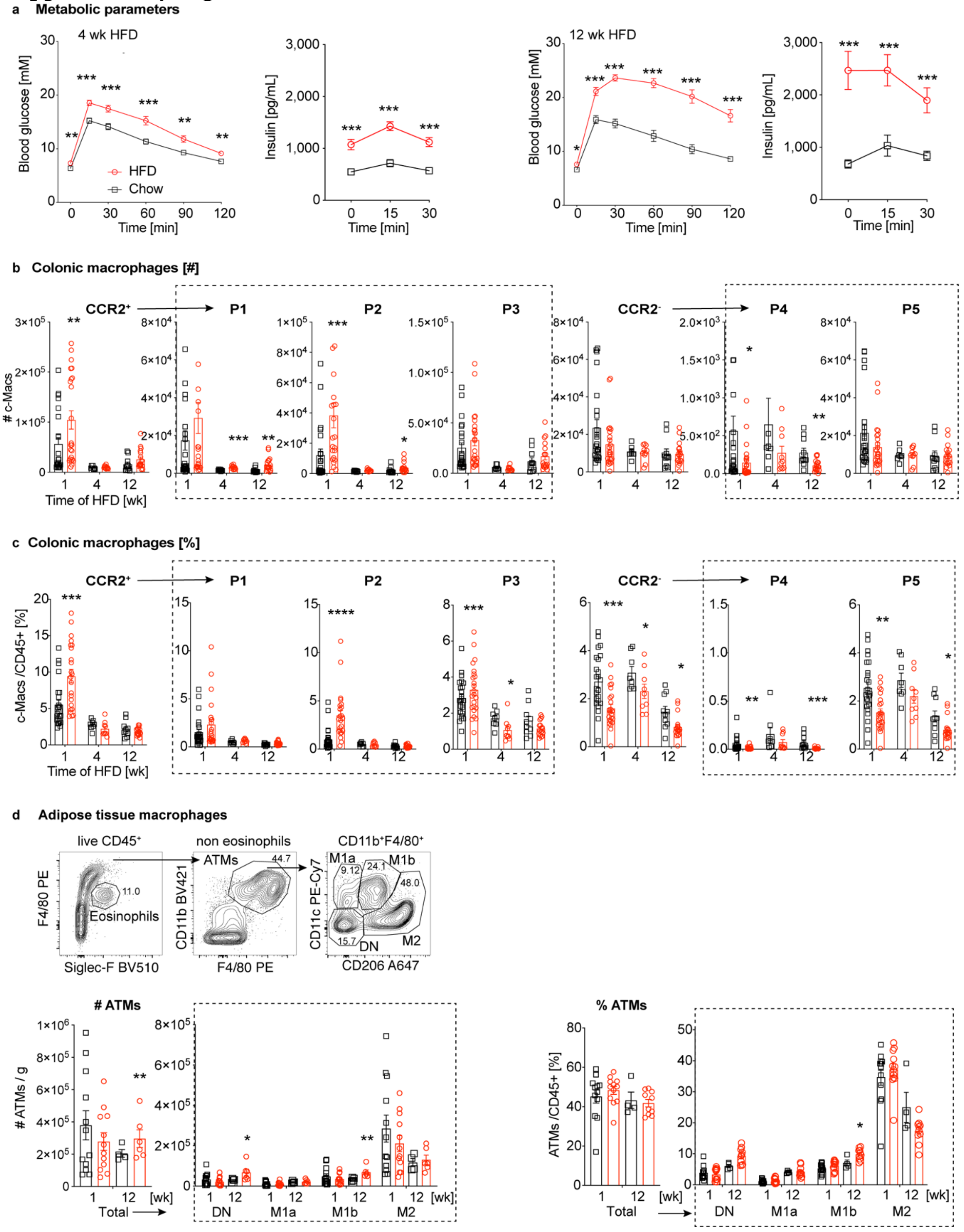

Supplementary Fig. 1. Early increase of colonic macrophages, followed later by increased adipose tissue macrophages after HFD feeding. Wild-type mice were fed either coconut-based HFD (red circles) or chow control diet (black squares) for up to 12 weeks: **a,** Intraperitoneal glucose tolerance test (IPGTT) and insulin (HFD/chow 4 weeks n=21/n=17, 12 weeks n=18/n=11). **b,c,** Absolute numbers (#) or frequency (%) of monocyte-derived $CCR2^+$ (pro-inflammatory P1, P2, intermediate P3 subpopulations) and anti-inflammatory/resident $CCR2^-$ colonic macrophages (c-Macs: P4, P5) . **d,** Flow cytometry gating strategy, absolute numbers per gram (#/g) and frequency of adipose tissue macrophages (ATMs: DN, M1a, M1b, M2). Statistical data are expressed as mean±SEM. Data are a representative of 2-6 independent experiments, with each data point representing one individual mouse. *$p<0.05$, **$p<0.01$, ***$p<0.001$, unpaired Mann-Whitney U test with two tailed distribution.

Supplementary Fig. 2

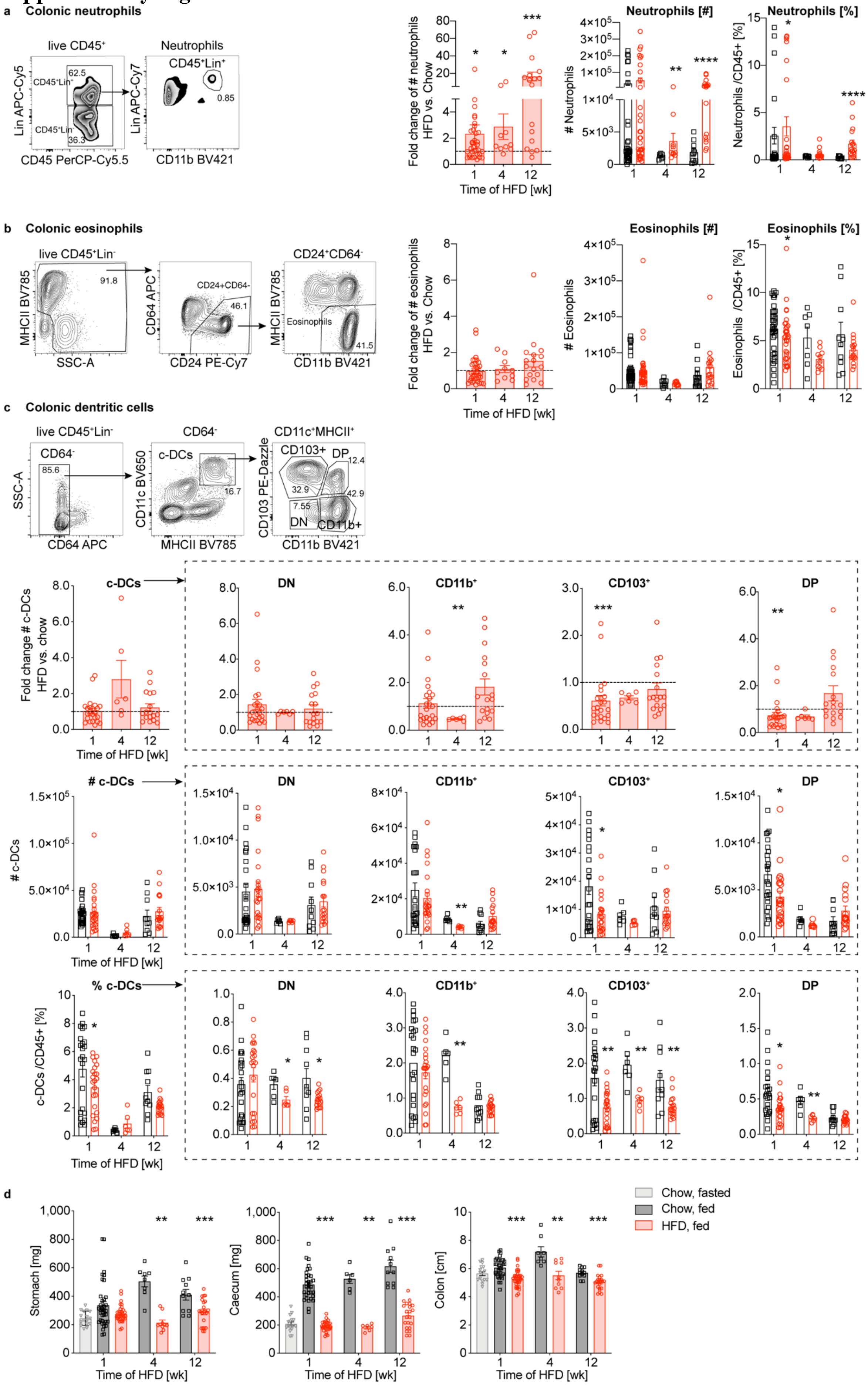

Supplementary Fig. 2. Increased colonic neutrophils, but no consistent pattern in colonic dendritic subpopulations upon HFD. Wild-type mice were fed either coconut-based HFD (red circles), or chow control diet (black squares) for up to 12 weeks: **a,b,** Flow cytometry gating strategy, fold change of absolute numbers (#), absolute numbers and frequency of colonic neutrophils (**a**) and eosinophils (**b**). **c,** Flow cytometry gating strategy, fold change of absolute numbers (#), absolute numbers and frequency of colonic dendritic cells (c-DCs) and their subpopulations (DN, $CD11b^+$, $CD103^+$, DP). **d,** Macroscopic measurements of stomach, caecum and colon in wild-type mice fasted (grey), fed chow (black) or HFD (red). Statistical data are expressed as mean±SEM. Data are a representative of 2-6 independent experiments, with each data point representing one individual mouse. *p<0.05, **p<0.01, ***p<0.001, unpaired Mann-Whitney U test with two tailed distribution.

Supplementary Fig. 3

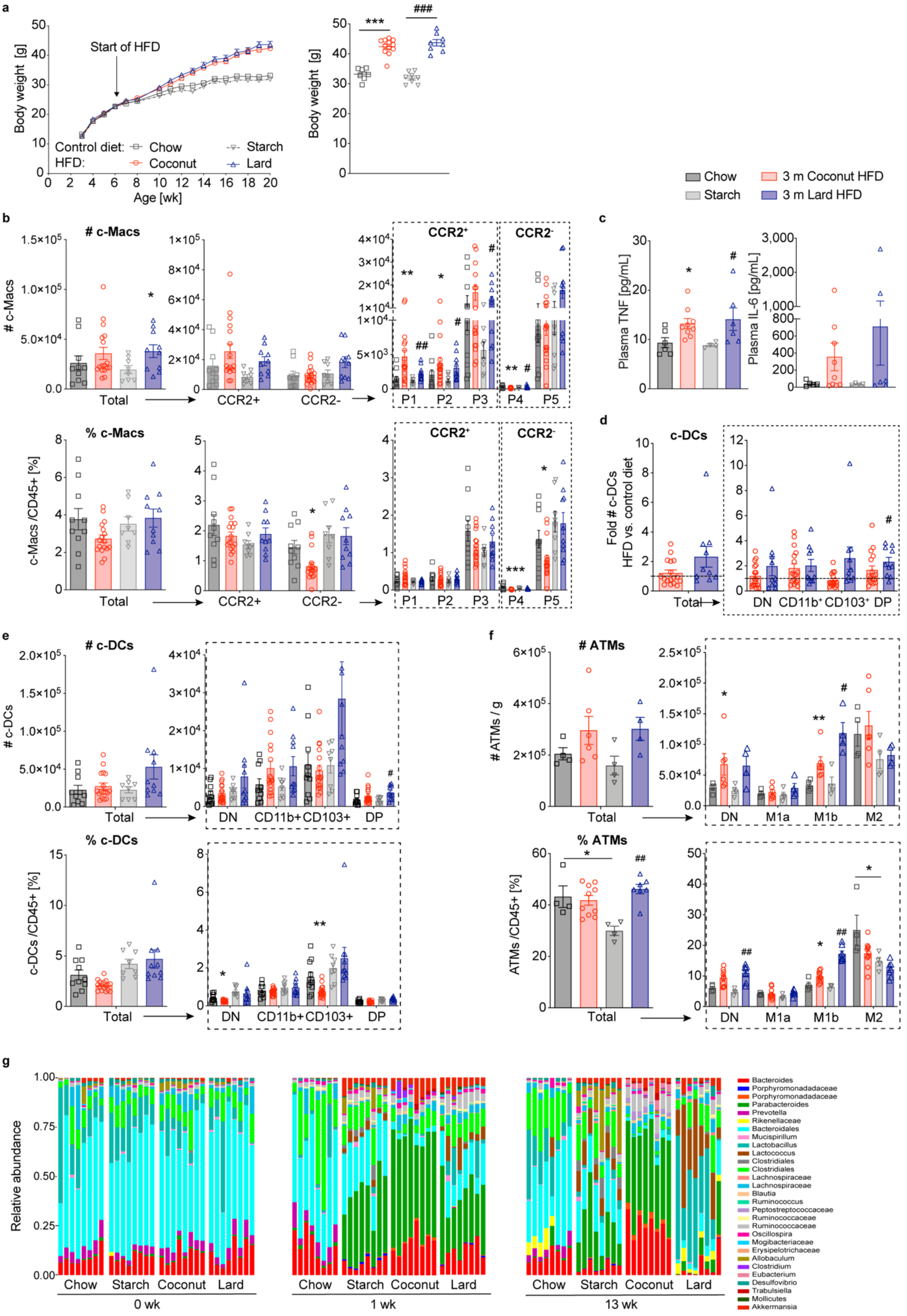

Supplementary Fig. 3. Changes in intestinal immunity in relation to fibers content, fat source and gut microbiota. Wild-type mice were fed 3 months (3 m) HFD (coconut: red circles or lard-based: blue triangles), or control diet (starch without fibers: gray triangles, chow with fibers: black squares): **a,** Body weights (coconut n=12, lard n=8, chow n=8, starch n=8). **b,** Absolute numbers (#) and frequency (%) of colonic macrophages (c-Macs). **c,** Plasma TNF and IL-6. **d,** Fold change of absolute numbers of colonic dendritic cells (c-DCs: DN, $CD11b^{+}$, $CD103^{+}$ DP) after 3 m HFD compared to respective control diets (coconut-HFD vs. chow, lard-HFD vs. starch). **e,** Absolute numbers (#) and frequencies of c-DCs. **f,** Absolute numbers (#/g) or frequencies of adipose tissue macrophages (ATMs). **g,** Relative genus abundances of fecal microbiota before and after 1 week (1 wk) and 3 m HFD, compared to controls (n=8 per group). Statistical data are expressed as mean±SEM. Data are representative of two independent experiments (**b,e,d**), or of one experiment (**a,c,f,g**), with each data point representing an individual mouse. Coconut-based HFD vs. chow: *$p<0.05$, **$p<0.01$, ***$p<0.001$. Lard-based HFD vs. starch: $^{\#}p<0.05$, $^{\#\#}p<0.01$, $^{\#\#\#}p<0.001$, unpaired Mann-Whitney U test with two tailed distribution. See also Supplementary Fig. 2.

54 **Supplementary Fig. 4**

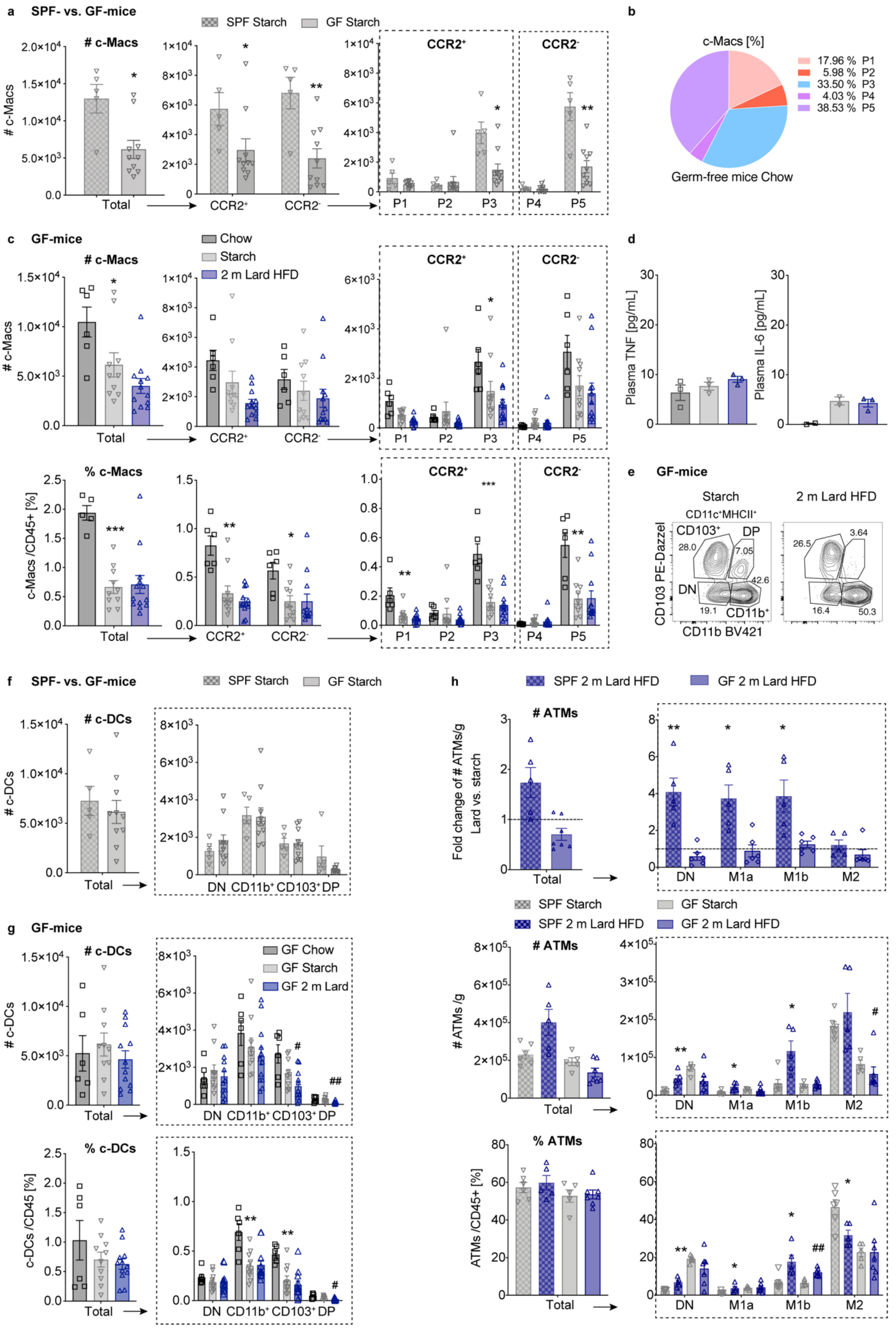

55

Supplementary Fig. 4. Changes in intestinal and adipose tissue macrophages in relation to fiber content and gut microbiota. Specific pathogen free (SPF) or germ-free (GF) mice were fed 2 months (2 m) lard-based HFD (blue triangles), or control diet (chow: black squares, starch: gray triangles): **a,** Absolute numbers (#) of colonic macrophages (c-Macs) in SPF- and GF-mice fed starch control diet. **b,** Distribution of P1-P5 c-Macs (**b**) in GF-mice fed chow (black n=6). **c,d** Absolute numbers (#) and frequency of c-Macs (**c**) as well as Plasma TNF and IL-6 (**d**) in GF-mice. **e,** Representative flow cytometry plot of colonic dendritic cells (c-DCs) in GF-mice. **f,** Absolute numbers of c-DCs in starch fed SPF-mice compared to GF-mice. **g,** Absolute numbers and frequency of c-DCs in GF-mice compared to chow or starch control diet. **h,** Fold change, absolute numbers per gram (#/g) and percentage of adipose tissue macrophages (ATMs) in HFD SPF- or GF-mice compared to their starch controls. Statistical data are expressed as mean±SEM. Data are a representative of one (**a,d,f,h** SPF in parallel to GF-mice) or 2-3 independent experiments (**b,c,g**), with each data point representing one individual mouse. **c,f:** Chow compared to starch fed GF-mice: *$p<0.05$, **$p<0.01$. Lard-based HFD vs. starch: $^{\#}p<0.05$, **h:** HFD SPF-mice vs. starch: *$p<0.05$, **$p<0.01$, ***$p<0.001$. HFD GF-mice vs. starch: $^{\#}p<0.05$, $^{\#\#}p<0.01$, unpaired Mann-Whitney U test with two tailed distribution.

Supplementary Fig. 5

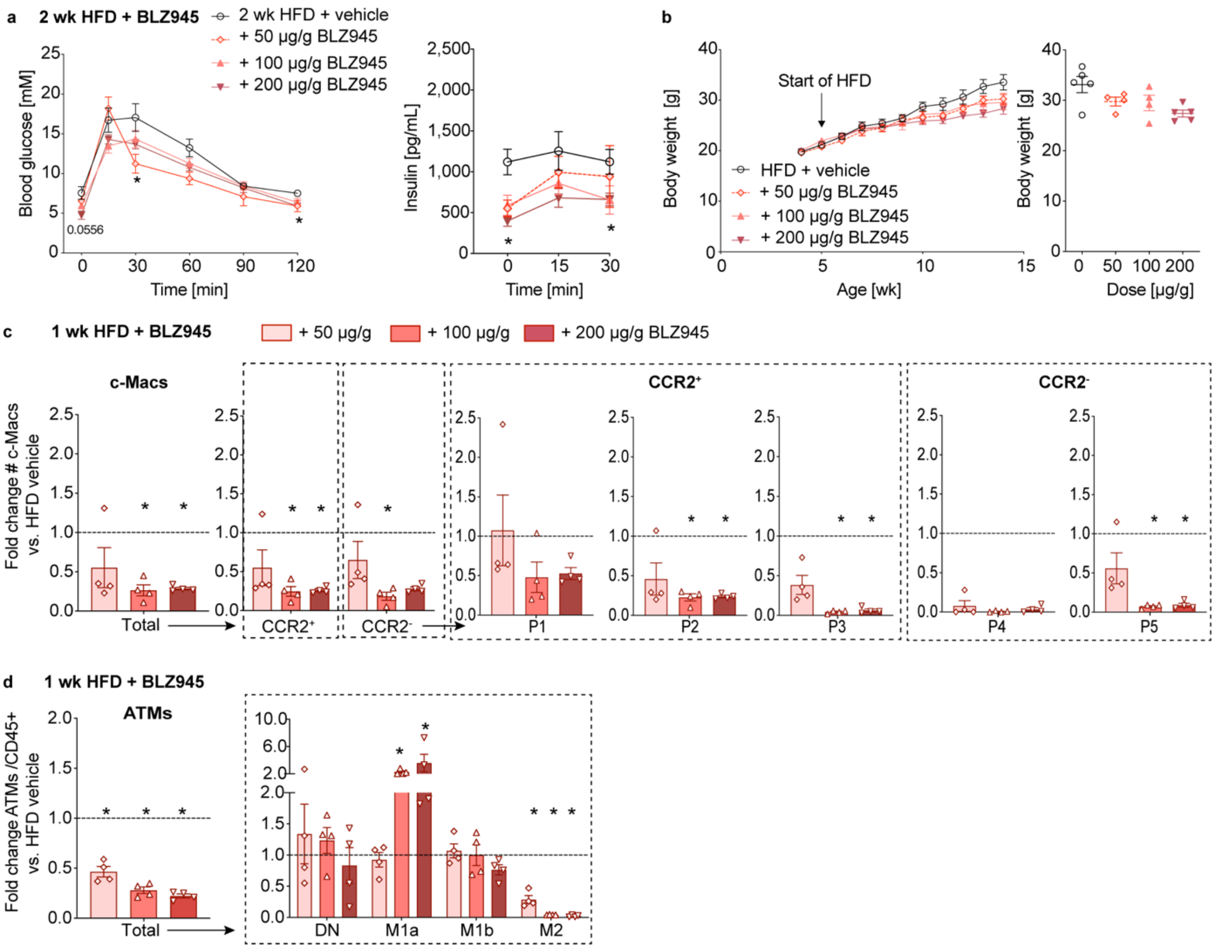

Supplementary Fig. 5. Systemic pharmacological and genetic depletion of macrophages improves glycemic control. Wild-type mice were fed 1-2 weeks (wk) coconut-based HFD and treated with the CSF1R inhibitor BLZ945 or vehicle (vehicle n=5: black circles, 50 µg/g n=4: light red rhombus, 100 µg/g n=4: light red triangles, 200 µg/g n=5: dark red triangles): **a,** Intraperitoneal glucose tolerance test (IPGTT), insulin after 2 wk HFD and 3 wk BLZ945 treatment. **b,** Body weight over 2 months of HFD and BLZ945 treatment. **c,d,** Fold change of colonic macrophages (c-Macs) (**c**) and adipose tissue macrophages (ATMs) (**d**) after 1 wk HFD and 2 wk oral BLZ945 treatment compared to vehicle treated mice. Statistical data are expressed as mean±SEM. Data are representative of one experiment, with each data point representing one individual mouse. *p<0.05, **p<0.01, unpaired Mann-Whitney U test with two tailed distribution.

Supplementary Fig. 6

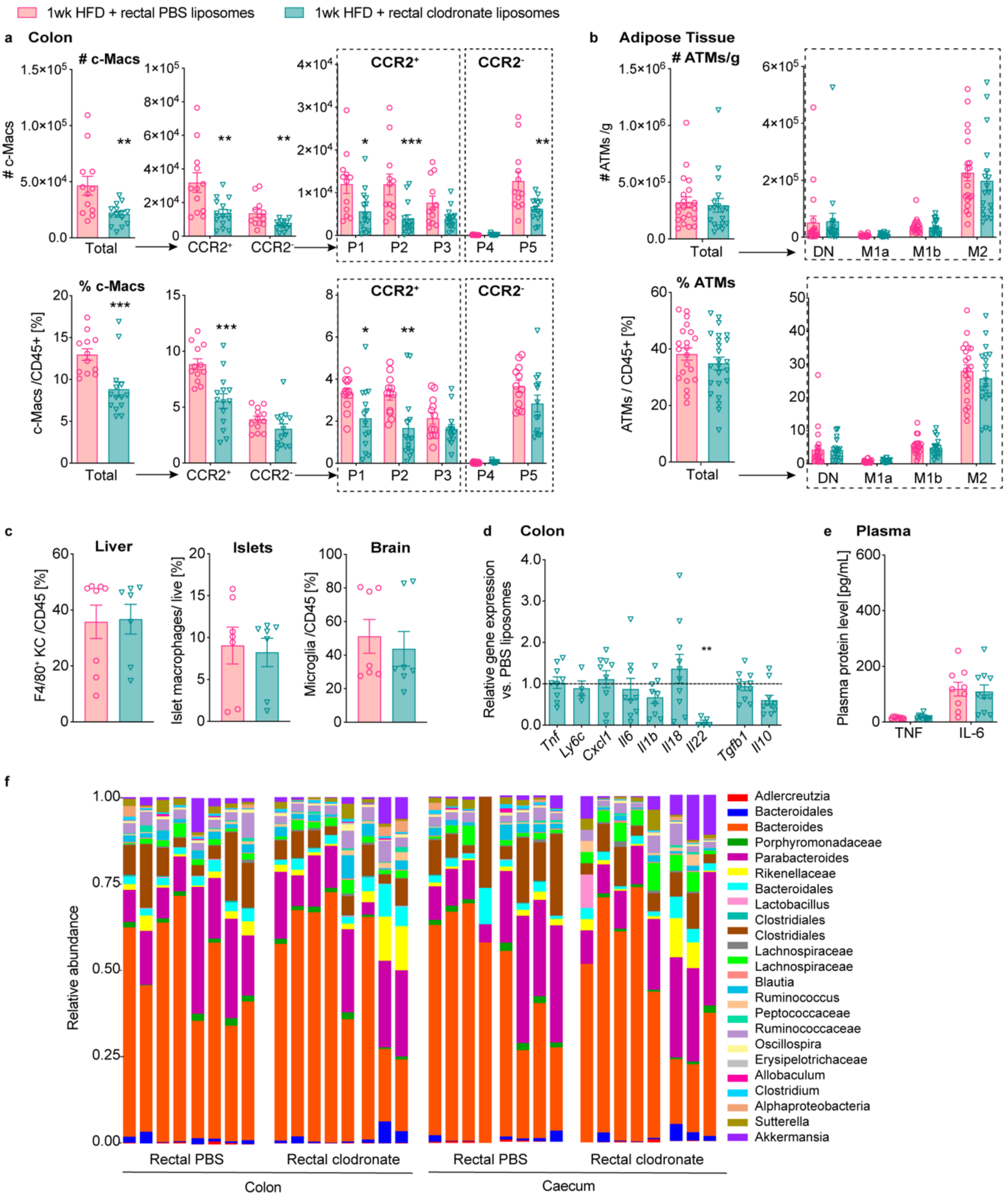

Supplementary Fig. 6. Effect of intrarectal clodronate liposomes upon HFD feeding. Wild-type mice were fed 1 week (wk) coconut-based HFD, and treated intrarectally with clodronate (turquoise triangles) or PBS liposomes (pink circles): **a,** Absolute numbers (#) and frequency of colonic macrophages (c-Macs) in the proximal colon. **b,** Absolute numbers per gram (#/g) and frequency of adipose tissue macrophages (ATMs). **c,** Frequency of Kupffer cells (KCs), islet macrophages, and microglia. **d,** Colon tissue gene expression of HFD mice treated intrarectally with clodronate liposomes relative to PBS controls. **e,** Plasma TNF and IL-6. **f,** Relative genus abundances of fecal microbiota (clodronate n=8, PBS n=8). Statistical data are expressed as mean±SEM. Data are a representative of tree (**a,b,e**), two (**c,d,f**) independent experiments, with each data point representing one individual mouse. *p<0.05, **p<0.01, ***p<0.001, unpaired Mann-Whitney U test with two tailed distribution.

101 **Supplementary Fig. 7**

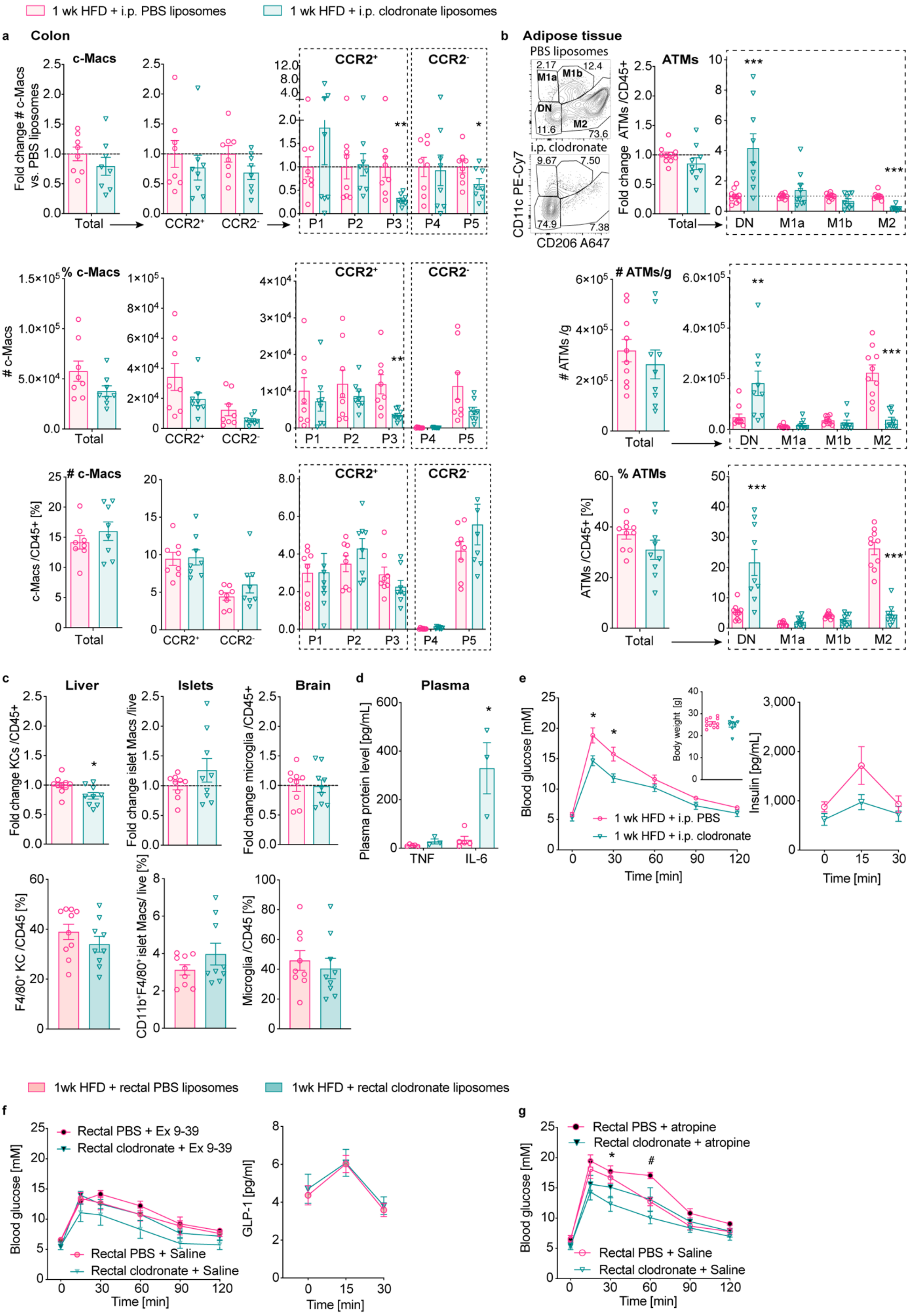

102

Supplementary Fig. 7. Effect of intraperitoneal and rectal clodronate liposomes under HFD. Wild-type mice were fed 1 week (wk) coconut-based HFD and intraperitoneally treated with clodronate (turquoise triangles) or PBS liposomes (pink circles): **a,** Fold change, absolute numbers (#) and frequencies of colonic macrophages (c-Macs) in the proximal colon. **b,** Representative flow cytometry plots and fold change, absolute numbers (#/g), and frequencies of adipose tissue macrophages (ATMs). **c,** Fold change and frequency of Kupffer cells (KCs), islet macrophages and microglia. **d,** Plasma TNF and IL-6. **e,** Intraperitoneal glucose tolerance test (IPGTT), body weight, and insulin (clodronate n=9, PBS liposomes n=10). Wild-type mice were fed 1 week (wk) coconut-based HFD and intrarectally treated with clodronate (turquoise) or PBS liposomes (pink): **f,** Oral GTT (OGTT) blocking GLP-1 action by exendin (9-39) (clodronate n=7, PBS liposomes n=8) compared to saline injected controls (clodronate n=5, PBS n=4) and GLP-1 secretion upon oral GTT. **g,** OGTT after atropine (clodronate n=7, PBS n=7) or saline (clodronate n=7, PBS n=6) injection. Statistical data are expressed as mean±SEM. Data are a representative of two (**a-c**) independent experiments and one (**d-g**) experiment, with each data point representing one individual mouse. *p<0.05, **p<0.01, unpaired Mann-Whitney U test with two tailed distribution.

Supplementary Fig. 8

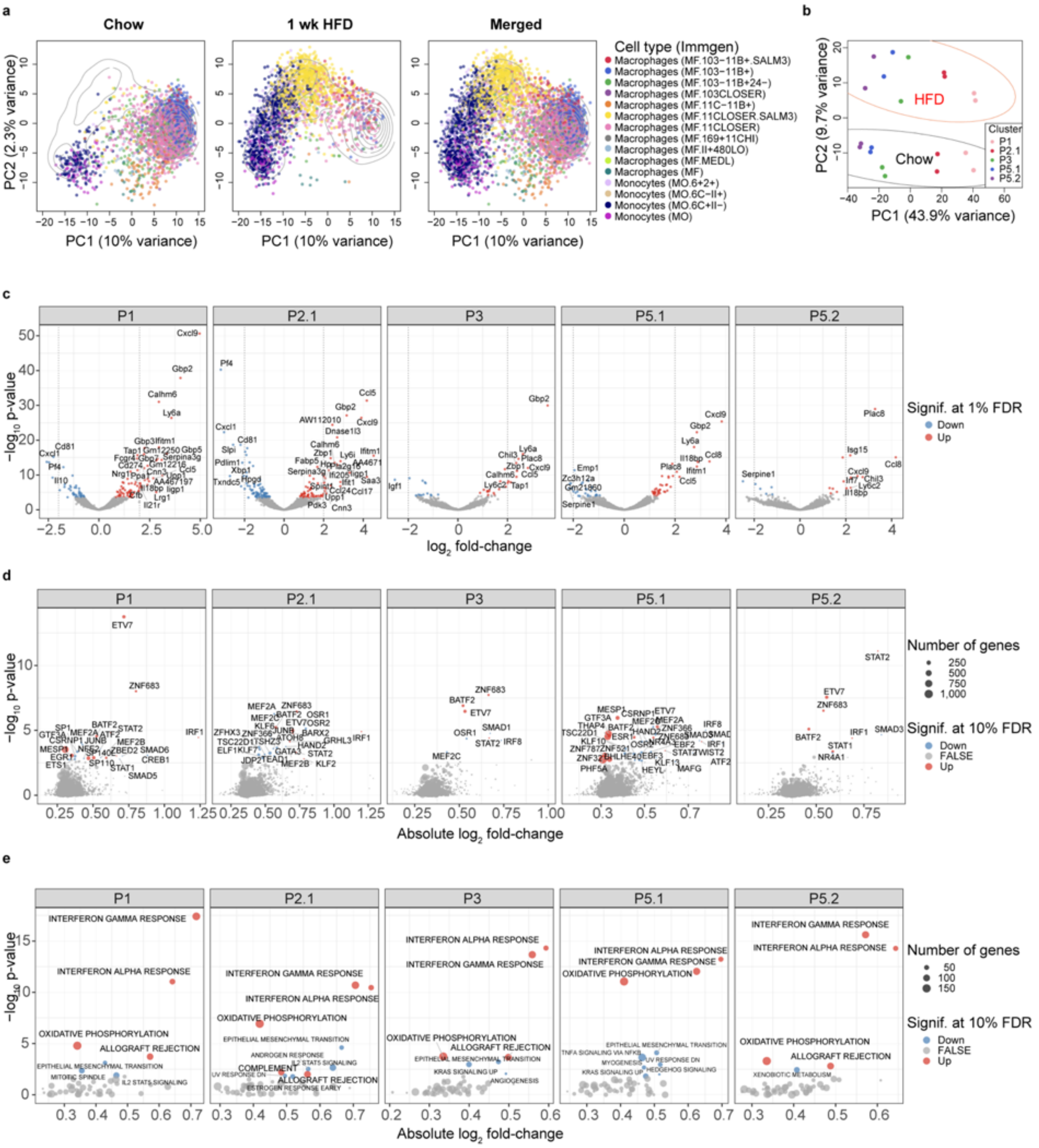

Supplementary Fig. 8. Transcriptional profiling of colonic macrophages after HFD. Single cell RNA-seq in wild-type mice fed 1 week (wk) coconut-based HFD, or control diet: **a,** Principal component analysis (PCA) of sorted colonic macrophages (c-Macs). One point represents one cell, colored by the annotated cell type using the ImmGen microarray reference (cell types with at least 10 cells annotated are shown). **b,** PCA of pseudo-bulk RNA-seq samples used for differential expression analysis between chow and HFD. One point represents one macrophage cluster per mouse. **c,** Volcano plots showing the differential expression between HFD and chow for each cluster. Up- (red) or down-regulated (blue) genes are shown when significant at 1% FDR threshold and absolute $\log_2$ fold-change above 2. **d,** Up- (red) or down-regulated (blue) regulons after HFD compared to chow for each cluster (10% FDR threshold). **e,** Up- (red) or down-regulated (blue) MSigDB hallmark pathways after HFD compared to chow for each cluster (10% FDR threshold). Data are representative of two replicates (n=2 per group) (**a-e**) and point sizes reflect the number of genes included in each annotated pathway (**d-e**).

Supplementary Fig. 9

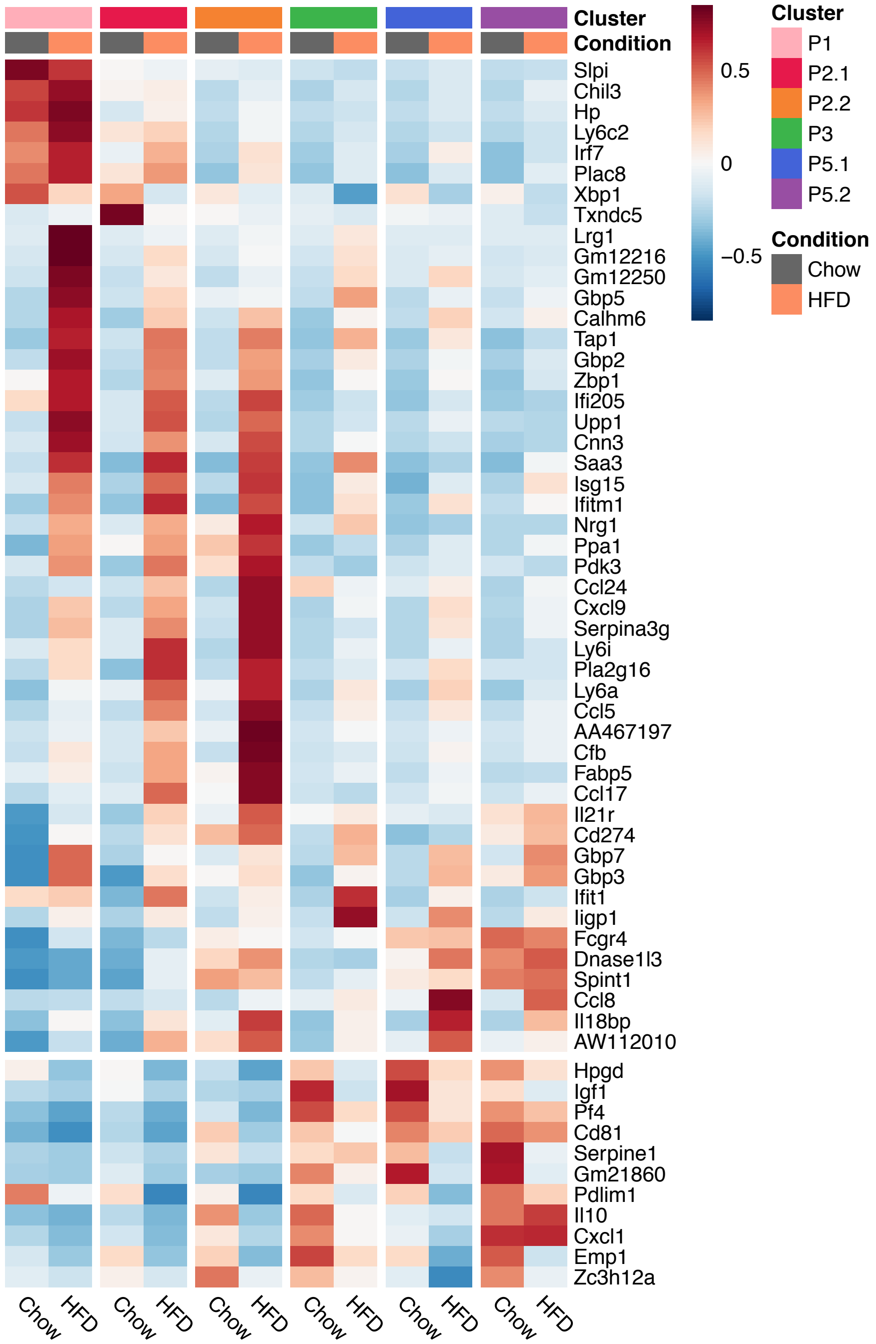

Supplementary Fig. 9. Differential gene expression in colonic macrophages upon HFD. Average expression of genes differentially expressed in colonic macrophages of mice fed 1 week HFD (n=2) vs. chow (n=2). Differential expression testing was done per cluster (P1, P2.1, P3, P5.1 and P5.2), significant at a 1% FDR threshold and with absolute $\log_2$ fold-change above 2. Genes overall up-regulated in HFD vs. chow are separated with a gap to those overall down-regulated.

Supplementary Fig. 10

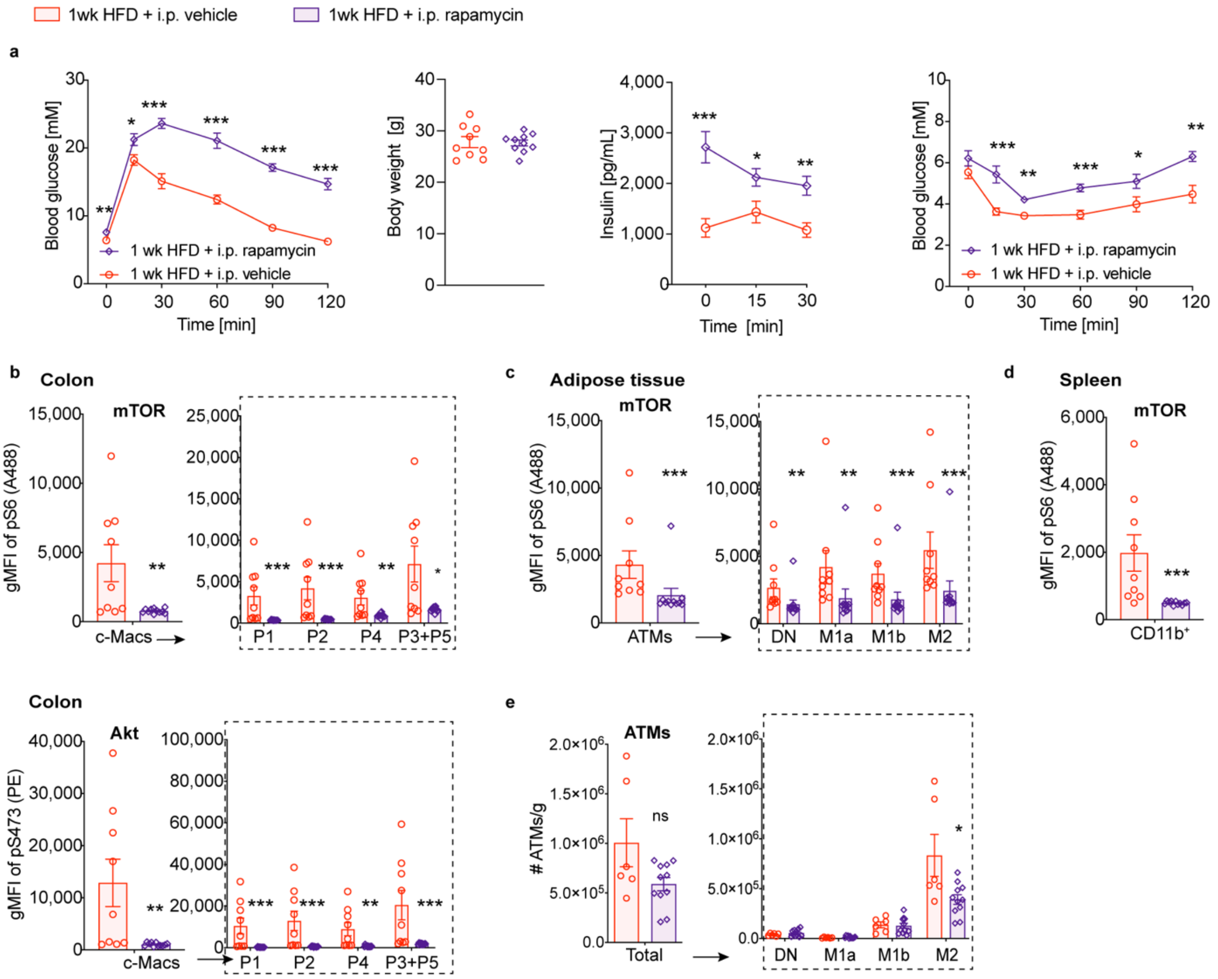

Supplementary Fig. 10. The effect of intraperitoneal rapamycin under HFD. Wild-type mice were fed 1 week (wk) coconut-based HFD and treated intraperitoneally with 3mg/kg rapamycin (purple rhombus) or vehicle (red circles): **a,** Intraperitoneal glucose tolerance test (IPGTT), body weight at IPGTT, insulin (rapamycin n=10, vehicle n=9), and insulin tolerance test (ITT: rapamycin n=9, vehicle n=9). **b,** Geometric mean fluorescent intensity (gMFI) of pS6 and pS473 in colonic macrophages (c-Macs). **c-e,** gMFI of pS6 in adipose tissue macrophages (ATMs) (**c**) and in splenic monocytes (**d**) and ATMs per gram (#ATMs/g) (**e**). Statistical data are expressed as mean±SEM. Data are a representative of one experiment, with each data point representing one individual mouse. *p<0.05, **p<0.01, ***p<0.001, unpaired Mann-Whitney U test with two tailed distribution.

Supplementary Fig. 11

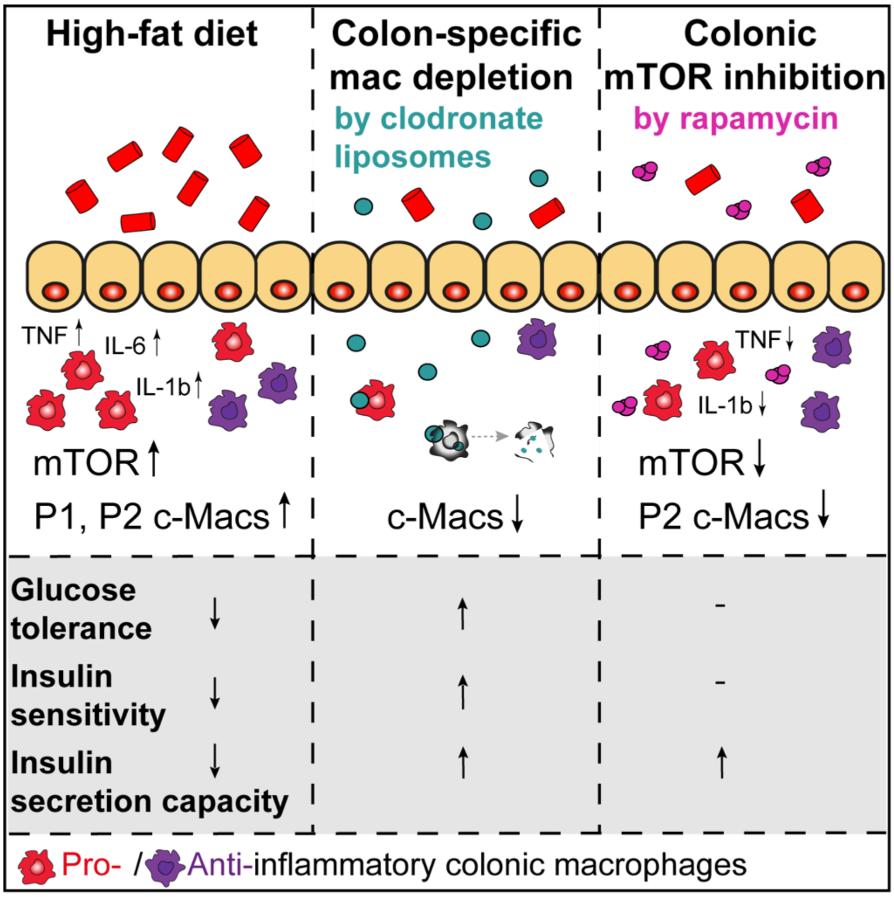

Supplementary Fig. 11: Summary of models tested: HFD leads to activation of mTOR and an increase in pro-inflammatory colonic macrophages, which is accompanied by glucose intolerance, reduced insulin sensitivity, and insulin secretion capacity. Colon-specific macrophage depletion by rectal clodronate liposomes improves glucose metabolism. Rectal rapamycin reduces mTOR activity and pro-inflammatory macrophages in the colon, resulting in a higher insulin secretion capacity.

Supplementary tables with titles and legends

Supplementary Table 1. Diet composition of control diets and HFD

Diet	Fat	Protein	Carbohydrates	Fibers
Coconut-based HFD	58 % Coconut fat (Coconut and soybean oil)	16.4 %	25.5 % (Maltodextrin 10, Sucrose)	0.5 %
Chow diet (control diet for coconut-based HFD)	4.5 % Crude fat	18.5 %	35 % Starch	4.5 %
Lard-based HFD	60 % Lard fat (Lard and soybean oil)	20 %	20 % (Maltodextrin 10, Sucrose)	0 %
Starch (control diet for lard-based HFD)	10 % Lard fat (Lard and soybean oil)	20 %	70 % Starch (Maltodextrin 10, Sucrose)	0 %
Olive-oil based HFD	50 % Olive oil	20.2%	22.3 % Sugar, 0.1% Starch	1.8 %
Coconut-oil based HFD (control diet for olive oil-based HFD)	50 % Coconut fat (Coconut and soybean oil)	20.2%	22.3% Sugar, 0.1% Starch	1.8 %

Units in kcal%.

Supplementary Table 2. Primers sequences used for quantitative RT-PCR

**Gene**	**Forward Primer**	**Reverse Primer**
**Housekeeping genes**		
B2m	5′ TTCTGGTGCTTGTCTCACTGA	5′ CAGTATGTTCGGCTTCCCATTC
Ppia	5′ GAGCTGTTTGCAGACAAAGTTC	5′ CCCTGGCACATGAATCCTGG
**Inflammation markers**		
Tnf	5′ ACTGAACTTCGGGGTGATCG	5′ TGAGGGTCTGGGCCATAGAA
Il6	5′ GGATACCACTCCCAACAGACCT	5′ GCCATTGCACAACTCTTTTCTC
Il1b	5′ GCAACTGTTCCTGAACTCAACT	5′ ATCTTTTGGGGTCCGTCAACT
Kc	5′ CTGGGATTCACCTCAAGAACATC	5′ CAGGGTCAAGGCAAGCCTC
Il10	5′ AGGCGCTGTCATCGATTTCTC	5′ GCCTTGTAGACACCTTGGTCTT
Il18	5′TCTTGCGTCAACTTCAAGGA	5′GTGAAGTCGGCCAAAGTTGT
Il22	5′TTG AGG TGT CCA ACT TCC AGC A	5′AGC CGG ACG TCT GTG TTG TTA
Tgfb1	5′CTCTCCACCTGCAAGACCAT	5′CGAGCCTTAGTTTGGACAGG
**Immune cells**		
Ly6c	5′ GCAGTGCTACGAGTGCTATGG	5′ ACTGACGGGTCTTTAGTTTCCTT
Cd68	5′ GCAGCACAGTGGACATTCAT	5′ AGAGAAACATGGCCC GAAGT
Emr1	5′ GCC CAG GAGTGGAATGTCAA	5′ CAGACACTCATCAACATCTGCG

Supplementary Table 3. Primers sequences used for 16S amplicon PCR

**1**	CTAAGGTAAC	**33**	TTCTCATTGAAC	**65**	TCCTGGCACATC
**2**	TAAGGAGAAC	**34**	TCGCATCGTTC	**66**	CCGCAATCATC
**3**	AAGAGGATTC	**35**	TAAGCCATTGTC	**67**	TTCCTACCAGTC
**4**	TACCAAGATC	**36**	AAGGAATCGTC	**68**	TCAAGAAGTTC
**5**	CAGAAGGAAC	**37**	CTTGAGAATGTC	**69**	TTCAATTGGC
**6**	CTGCAAGTTC	**38**	TGGAGGACGGAC	**70**	CCTACTGGTC
**7**	TTCGTGATTC	**39**	TAACAATCGGC	**71**	TGAGGCTCCGAC
**8**	TTCCGATAAC	**40**	CTGACATAATC	**72**	CGAAGGCCACAC
**9**	TGAGCGGAAC	**41**	TTCCACTTCGC	**73**	TCTGCCTGTC
**10**	CTGACCGAAC	**42**	AGCACGAATC	**74**	CGATCGGTTC
**11**	TCCTCGAATC	**43**	CTTGACACCGC	**75**	TCAGGAATAC
**12**	TAGGTGGTTC	**44**	TTGGAGGCCAGC	**76**	CGGAAGAACCTC
**13**	TCTAACGGAC	**45**	TGGAGCTTCCTC	**77**	CGAAGCGATTC
**14**	TTGGAGTGTC	**46**	TCAGTCCGAAC	**78**	CAGCCAATTCTC
**15**	TCTAGAGGTC	**47**	TAAGGCAACCAC	**79**	CCTGGTTGTC
**16**	TCTGGATGAC	**48**	TTCTAAGAGAC	**80**	TCGAAGGCAGGC
**17**	TCTATTCGTC	**49**	TCCTAACATAAC	**81**	CCTGCCATTCGC
**18**	AGGCAATTGC	**50**	CGGACAATGGC	**82**	TTGGCATCTC
**19**	TTAGTCGGAC	**51**	TTGAGCCTATTC	**83**	CTAGGACATTC
**20**	CAGATCCATC	**52**	CCGCATGGAAC	**84**	CTTCCATAAC
**21**	TCGCAATTAC	**53**	CTGGCAATCCTC	**85**	CCAGCCTCAAC
**22**	TTCGAGACGC	**54**	CCGGAGAATCGC	**86**	CTTGGTTATTC
**23**	TGCCACGAAC	**55**	TCCACCTCCTC	**87**	TTGGCTGGAC
**24**	AACCTCATTC	**56**	CAGCATTAATTC	**88**	CCGAACACTTC
**25**	CCTGAGATAC	**57**	TCTGGCAACGGC	**89**	TCCTGAATCTC
**26**	TTACAACCTC	**58**	TCCTAGAACAC	**90**	CTAACCACGGC
**27**	AACCATCCGC	**59**	TCCTTGATGTTC	**91**	CGGAAGGATGC
**28**	ATCCGGAATC	**60**	TCTAGCTCTTC	**92**	CTAGGAACCGC
**29**	TCGACCACTC	**61**	TCACTCGGATC	**93**	CTTGTCCAATC
**30**	CGAGGTTATC	**62**	TTCCTGCTTCAC	**94**	TCCGACAAGC
**31**	TCCAAGCTGC	**63**	CCTTAGAGTTC	**95**	CGGACAGATC
**32**	TCTTACACAC	**64**	CTGAGTTCCGAC	**96**	TTAAGCGGTC
