# Supplementary material for: Targeting colonic macrophages improves glycemic control in high-fat diet-induced obesity
Source: Commun Biol. 2022 Apr 19;5:370. doi: 10.1038/s42003-022-03305-z (PMC9018739; doi:10.1038/s42003-022-03305-z)
Supplement: Supplementary file 3 — Description of Additional Supplementary Files [file 42003_2022_3305_MOESM3_ESM.pdf]

Description of Additional Supplementary Files

File name: Supplementary Data 1
Description: Source data of main figures.

File name: Supplementary Data 2
Description: Key resources table.
