# Supplementary material for: Targeting colonic macrophages improves glycemic control in high-fat diet-induced obesity
Source: Commun Biol. 2022 Apr 19;5:370. doi: 10.1038/s42003-022-03305-z (PMC9018739; doi:10.1038/s42003-022-03305-z)
Supplement: Supplementary file 6 — Reporting Summary [file 42003_2022_3305_MOESM6_ESM.pdf]

nature portfolio

Corresponding author(s): DBPR, #NCOMMS-21-38734

Last updated by author(s): Feb 5, 2022

Reporting Summary

Nature Portfolio wishes to improve the reproducibility of the work that we publish. This form provides structure for consistency and transparency in reporting. For further information on Nature Portfolio policies, see our Editorial Policies and the Editorial Policy Checklist.

Statistics

For all statistical analyses, confirm that the following items are present in the figure legend, table legend, main text, or Methods section.

n/a	Confirmed	
☐	☒	The exact sample size (n) for each experimental group/condition, given as a discrete number and unit of measurement
☐	☒	A statement on whether measurements were taken from distinct samples or whether the same sample was measured repeatedly
☐	☒	The statistical test(s) used AND whether they are one- or two-sided<br>*Only common tests should be described solely by name; describe more complex techniques in the Methods section.*
☒	☐	A description of all covariates tested
☒	☐	A description of any assumptions or corrections, such as tests of normality and adjustment for multiple comparisons
☒	☐	A full description of the statistical parameters including central tendency (e.g. means) or other basic estimates (e.g. regression coefficient) AND variation (e.g. standard deviation) or associated estimates of uncertainty (e.g. confidence intervals)
☒	☐	For null hypothesis testing, the test statistic (e.g. F, t, r) with confidence intervals, effect sizes, degrees of freedom and P value noted<br>*Give P values as exact values whenever suitable.*
☒	☐	For Bayesian analysis, information on the choice of priors and Markov chain Monte Carlo settings
☒	☐	For hierarchical and complex designs, identification of the appropriate level for tests and full reporting of outcomes
☒	☐	Estimates of effect sizes (e.g. Cohen's d, Pearson's r), indicating how they were calculated

Our web collection on statistics for biologists contains articles on many of the points above.

Software and code

Policy information about availability of computer code

Data collection	DB FACSDiva v 8.0.1. NextSeq Control Software (NCS, Illumina), NanoDrop, ViiA7 Real-Time PCR System (Thermo Fisher Scientific), Ion Personal Genome Machine System (ThermoFisher).<br>See more details in software and algorithms section in Extended Data Table 1 with references and website information.
Data analysis	Microsoft Excel v16.35, GraphPad Prism v8.3.0, FlowJo v10.6.1, Fast QC tool v0.39.2, Kallisto v0.46.0, BUStool v 0.39.2, Ensembl release 97, Biodonductor 3.10 pakages, DorbletUtils v1.6.1, scran v1.14.5, scater v1.14.5, R v3.6, Single R v1.0.0, edge R v3.28, QIME pipline v1.8.0. Usearch61_ref v6.1.544, MaAsLin2, phyloseq. See more details in method section and Extended Data Table.1.

For manuscripts utilizing custom algorithms or software that are central to the research but not yet described in published literature, software must be made available to editors and reviewers. We strongly encourage code deposition in a community repository (e.g. GitHub). See the Nature Portfolio guidelines for submitting code & software for further information.

Data

Policy information about availability of data

All manuscripts must include a data availability statement. This statement should provide the following information, where applicable:

- Accession codes, unique identifiers, or web links for publicly available datasets
- A description of any restrictions on data availability
- For clinical datasets or third party data, please ensure that the statement adheres to our policy

The data availability statement is included in the manuscript, stating that sc-RNA-eq data are available in Gene Expression Omnibus (GEO), with the accession number GSE143351. Data sets generated during this study are available at https://www.ncbi.nlm.nih.gov/geo/query/acc.cgi?acc=GSE143351 by entering token unoveyewbnyzpuj into the search box.

Field-specific reporting

Please select the one below that is the best fit for your research. If you are not sure, read the appropriate sections before making your selection.

☒ Life sciences ☐ Behavioural & social sciences ☐ Ecological, evolutionary & environmental sciences

For a reference copy of the document with all sections, see nature.com/documents/nr-reporting-summary-flat.pdf

Life sciences study design

All studies must disclose on these points even when the disclosure is negative.

Sample size	The sample size was not predetermined and no formal power calculations were performed.
Data exclusions	Data was not excluded from the study, except because of technical issues during experiment resulting in low quality results not meeting the quality criteria. For sc-RNA-seq. analysis based on the distributions observed across cells, cells with library sizes lower than 795, total number of features detected lower than 317, or with a fraction of UMI counts attributed to the mitochondrial genes of 0 % or higher than 7 % were filtered out. Low-abundance genes with average normalized log2 counts lower than 0.003 were filtered out. For differential expression testing Cluster P2.2 was excluded because it contained too few chow cells. To analyze 16 S data, samples with fewer than 1,000 reads were excluded. See details in method sections.
Replication	Data of experiments were only considered in this study after method optimization and setting up a standard experimental procedure. The number of biological replicates is indicated in the figure legends, where each data point represents one individual mouse or subject. Only biological replicates are shown in this study, and no technical ones.
Randomization	Mice were randomized into different groups according to their starting weights.
Blinding	NA

Reporting for specific materials, systems and methods

We require information from authors about some types of materials, experimental systems and methods used in many studies. Here, indicate whether each material, system or method listed is relevant to your study. If you are not sure if a list item applies to your research, read the appropriate section before selecting a response.

Materials & experimental systems

n/a	Involved in the study	
☐	☒	Antibodies
☒	☐	Eukaryotic cell lines
☒	☐	Palaeontology and archaeology
☐	☒	Animals and other organisms
☒	☐	Human research participants
☒	☐	Clinical data
☒	☐	Dual use research of concern

Methods

n/a	Involved in the study	
☒	☐	ChIP-seq
☐	☒	Flow cytometry
☒	☐	MRI-based neuroimaging

Antibodies

Antibodies used	A complete list of used antibodies of this study is provided in Extended Data Table 1.
Validation	FACS antibodies were titrated. After validation concentration with highest staining index were used for this study.

Animals and other organisms

Policy information about studies involving animals; ARRIVE guidelines recommended for reporting animal research

Laboratory animals	Male C57BL/6N (Charles River Laboratories RRID:IMSR_CRL:027), male C57BL/6N (University of Basel RRID:IMSR_CRL:027), B6.129S4-Ccr2tm1Ifc/J (The Jackson Laboratory RRID:IMSR_JAX:004999), C57BL/6J mice (University of Basel and Bern RRID:IMSR_JAX:000664).
Wild animals	This study did not involve wild animals.
Field-collected samples	This study did not involve field-collected samples.
Ethics oversight	All animal procedures were approved by the local Animal Care and Use Committee and performed in accordance with Swiss Federal

Ethics oversight	regulations.

Note that full information on the approval of the study protocol must also be provided in the manuscript.

Flow Cytometry

Plots

Confirm that:

☒ The axis labels state the marker and fluorochrome used (e.g. CD4-FITC).

☒ The axis scales are clearly visible. Include numbers along axes only for bottom left plot of group (a 'group' is an analysis of identical markers).

☒ All plots are contour plots with outliers or pseudocolor plots.

☒ A numerical value for number of cells or percentage (with statistics) is provided.

Methodology

Sample preparation	Described in detail in method section.
Instrument	Flow cytometry analysis was performed with a BD LSRIIFortessa (BD) and sorting with BD FACS Aria III.
Software	BD FACSDiva (version 8.0.1), FlowJo (version 10.6.1)
Cell population abundance	For single cell-RNA-sequencing (scRNA-seq) (liveCD45+Lin-CD11b+CD24- and CD64+ or Ly6C+) CD11b+nonDCs colonic macrophages (see gating strategy7 and Fig. 1a) were sorted from mice fed 1 week HFD (n=2) or chow diet (n=2) by using FACS Aria III (BD Biosciences). See details in method section.
Gating strategy	See details in method section with links to figures showing gating strategy and references.

☒ Tick this box to confirm that a figure exemplifying the gating strategy is provided in the Supplementary Information.
